# Food insecurity during COVID-19 in children with end-stage kidney disease: a pilot study

**DOI:** 10.1186/s12887-022-03472-2

**Published:** 2022-07-15

**Authors:** Melvin Chan, Reya Mokiao, Amy C. Wilson, Neha Pottanat, Sangeeta Hingorani, Michelle C. Starr

**Affiliations:** 1 Division of Pediatric Nephrology, Department of Pediatrics, Indiana University School of Medicine and Riley Children’s Health, Indianapolis, IN USA; 2 Department of Pediatrics, University of Washington, Seattle, WA USA; 3 Indiana University School of Medicine, Health Information & Translational Sciences, 410 W 10th Street, Suite 2000A, Indianapolis, 46202 IN USA

**Keywords:** Coronavirus disease 2019, Food insecurity, Kidney disease, End-stage renal disease, Nutritional status

## Abstract

**Background:**

Food insecurity, an important social determinant of health among children, has become more common during the COVID-19 pandemic. Children with chronic diseases including end-stage kidney disease (ESKD) are at higher risk of food insecurity due to their complex care needs, medication burden, and dietary restrictions. No data exists describing food insecurity prevalence in pediatric ESKD patients during the COVID-19 pandemic.

**Methods:**

Food insecurity was assessed among families of children (age 0–18 years) with ESKD on chronic dialysis at two pediatric academic medical centers. Families were screened in April 2020 using the Hunger Vital Sign, a validated 2-question screening tool. We assessed impact of COVID-19 on food insecurity. We compared serum phosphorus “pre-COVID” (January/February 2020) to “during COVID” (April/May 2020).

**Results:**

A total of 29 families enrolled in this study. 62% (18/29) of children with ESKD lived in food insecure households, and of those, 72% (13/18) reported that COVID-19 had worsened their food insecurity status. During the COVID-19 pandemic, food insecure patients experienced greater rise in their serum phosphorus levels (1.1 mg/dL vs. 0 mg/dL, *p* = 0.03) and decreased likelihood of having adequate phosphorus control (50% vs. 11%, *p* = 0.03).

**Conclusion:**

Food insecurity was common among children with ESKD on chronic dialysis during the COVID-19 pandemic. Children with food insecurity had a greater increase in their phosphorus levels during the pandemic than did food secure children. Further exploration into how food resources such as an onsite food pantry impacts food insecurity and phosphorus control in children with ESKD is essential.

**Supplementary Information:**

The online version contains supplementary material available at 10.1186/s12887-022-03472-2.

## Background

Food insecurity (FI) is defined as limited or uncertain availability of nutritionally adequate and safe foods, or limited ability to acquire such food in socially acceptable ways [1]. Sixteen million households in the United States were food insecure in 2020, with 22% of American children living in food insecure households [1]. FI is an important social determinant of health and is more common in children with chronic diseases [2, 3]. Pediatric FI impacts a higher proportion of children with kidney disease, occurring in 35% of children seen in a general nephrology clinic and 65% of children with end-stage kidney disease (ESKD) [4, 5]. In children with ESKD, FI is associated with increased healthcare utilization and decreased quality of life [4, 5]. FI in children with kidney disease is also associated with higher phosphorus levels, as phosphorus content is often higher in low-cost shelf-stable processed foods [4].

During the COVID-19 pandemic, rates of FI have increased. Estimates suggest that during the height of the pandemic, FI rates tripled [6]. However, the impact of the pandemic on FI in children with chronic disease, such as kidney disease, is unknown. Evaluating the impact of the COVID-19 pandemic on food insecurity rates in children with chronic disease is paramount as a first step in connecting families to necessary community resources. In this study, we assessed the prevalence of FI among pediatric patients with ESKD during COVID-19. We hypothesized that the rates of FI in children with ESKD would be high during the COVID-19 pandemic, and that FI would correlate with worsening control of serum phosphorus, which may reflect quantity and quality of available food.

## Methods

We performed a pilot cohort study of pediatric patients with ESKD undergoing chronic hemodialysis (HD) or peritoneal dialysis (PD) during the COVID-19 pandemic at two institutions: Riley Hospital for Children at Indiana University (Indianapolis, Indiana) and Seattle Children’s Hospital (Seattle, Washington). Riley Hospital for Children is a free standing 354 bed pediatric hospital with 8 hemodialysis stations in the outpatient hemodialysis unit which serves a large catchment area and is the only dedicated pediatric hemodialysis unit in Indiana. Seattle Children’s Hospital is a 407 bed pediatric hospital with 7 hemodialysis stations in the outpatient hemodialysis unit. Patient characteristic and demographic data were gathered from the electronic medical record, including patient reported race, age, sex, and reason for ESKD.

Patients and families at both sites were screened for FI in April 2020 using the Hunger Vital Sign screen [7]. Consent was obtained from parents/guardians at Seattle Children’s Hospital prior to enrollment, and the study was deemed non-human subject research at Riley Hospital for Children and therefore consent was not required nor obtained. The Hunger Vital sign is a two-question tool validated in a variety of clinical settings, including the outpatient nephrology clinic and dialysis unit [4, 5, 7]. All families were screened using a paper copy of the Hunger Vital sign tool [5]. Families were included regardless of primary language and certified medical interpreters were utilized for all families where English was not their primary language. Families were identified as food insecure if they answered either “Often True” or “Sometimes True” to either of the two statements*: 1) Within the past 12 months we worried whether our food would run out before we got money to buy more* and *2) Within the past 12 months the food we bought just didn’t last and we didn’t have money to get more* [7]. If participants responded “Never True” to both Hunger Vital Sign questions, they were considered food secure.

We additionally assessed the impact of the COVID-19 pandemic on food security status with the following statement: *Because of the COVID Pandemic, access to food has been more challenging than it normally is [yes/no].* Food insecure families were referred to both short-term resources within the hospital, and connected with community-based resources to establish long-term supports. Of note, families with FI at Seattle Children’s Hospital had access to an on-site food pantry with ESKD appropriate foods, beginning in 2018, as previously described [4, 5]. Additionally, FI screening results from Seattle Children’s Hospital were compared prior FI prevelance data in previously published cohort of dialysis patients [4]. No prior FI prevalence data was available at Riley Hospital for Children.

We collected data on serum phosphorus levels as a surrogate marker of access to and intake of food as as phosphorus is often elevated in lower-cost, higher-processed shelf-stable foods [4]. We defined goal range serum phosphorus levels using laboratory age-based norms which mirror the KDIGO targets for normal phosphorus in children with ESKD [8]. We compared relevant clinical parameters “pre-COVID” (January/February 2020) to “during COVID” (April/May 2020) for each subject to assess changes over time during the COVID pandemic.

We summarized categorical variables by number and percentage, and continuous variables by mean and standard deviation. Comparisons between categorical variables was performed using Chi square or Fisher exact testing where appropriate and continuous variables were performed Wilcoxon’s rank sum, respectively. Statistical analyses were performed using Stata/SE 17.0 (StataCorp, College Station, Texas) and figures using Prism (Graphpad, San Diego, California). This study was approved by Institutional Review Boards at both institutions.

## Results

**Fig. 1:**
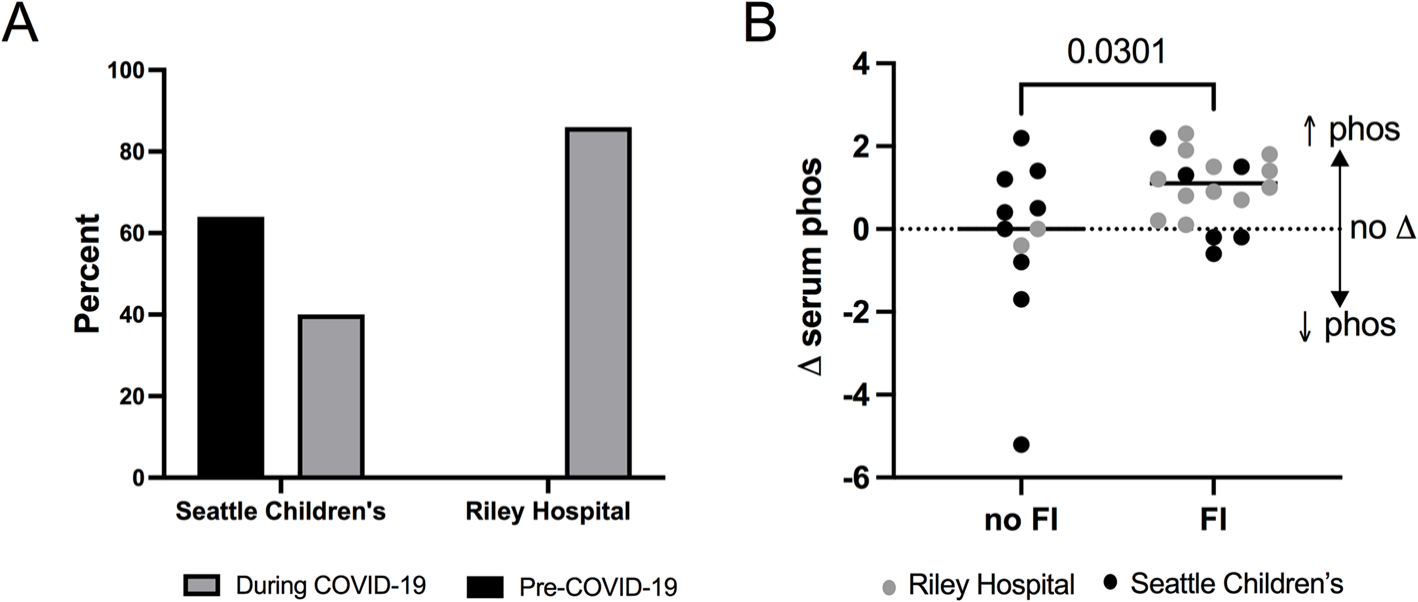
**A** Prevalence of food insecurity at Seattle Children’s Hospital and Riley Hospital for Children; 2019 pre-pandemic food insecurity prevalence at Seattle Children’s is included for context (note that no similar pre-COVID pandemic data available for Riley Hospital for Children, ND = No Data). **B** Change in serum phosphorus (expressed as Δ serum phosphorus, in mg/dL) from before COVID-19 (January/February 2020) to during COVID-19 (April/May 2020) comparing those without food insecurity (no FI) to those with food insecurity (FI)

A total of 29 families enrolled in this study (Supplemental Table 1). The majority of patients were on hemodialysis (86%), as at the time of the study Riley Hospital for Children only assessed FI in their hemodialysis patients. Eighteen children (62%) with ESKD were food insecure. There were no significant differences between demographic characteristics between food secure and food insecure participants (Supplemental Table 1). Children with ESKD at Riley Hospital for Children included this study were more likely to be male than those at Seattle Children’s (79% vs. 33%, *p* = 0.014). Otherwise, there were no significant differences in demographic characteristics between the two sites (Supplemental Table 2). At Seattle Children’s Hospital, which had previously performed FI screening, and where dialysis patients had access to an on-site food pantry, FI rates were significantly lower during COVID-19 (6/15 subjects or 40%) than in 2019 in a different clinical cohort of dialysis patients (28/44 subjects or 64%, *p* = 0.03). A higher percentage of study participants at Riley Hospital for Children were food insecure during the COVID-19 pandemic compared to Seattle Children’s Hospital (86% versus 40%, *p* = 0.01) (Fig. 1, Panel A). Among food insecure families, 13 (72%) reported that the COVID-19 pandemic had worsened their FI.

Children with ESKD who lived in food insecure households had a greater change in their serum phosphorus during COVID-19 than did food secure participants. Among children with FI, there was a median increase of 1.1 mg/dL [IQR -1.3] in serum phosphorus compared to a median change of 0 mg/dL [IQR 2] in the food secure group (*p* = 0.03, Fig. 1, Panel B, Supplemental Table 3). While not statistically significant, the median increase in serum phosphorus among food insecure children at Riley Hospital for Children was larger (1.1 mg/dL) than at Seattle Children’s Hospital (0.55 mg/dL). During COVID-19, food insecure patients were also less likely to have a serum phosphorus within goal range (decreasing from 50% before COVID-19 to 11% during COVID-19, *p* = 0.03). Among food secure children, there was no observed change in percentage with phosphorous values within goal range. (Supplemental Table 4).

## Discussion

The majority of children with ESKD on chronic dialysis participating in this study lived in food insecure households, and a majority of food insecure families reported worsening of their food insecurity due to the COVID-19 pandemic. We also found that children with FI had increasing serum phosphorus levels during the COVID-19 pandemic.

Children with ESKD may be at higher risk of FI given their frequent healthcare utilization and high medical expenditures. These risk factors may be exacerbated in the setting of a global pandemic [9]. Our finding of increasing serum phosphorus levels during COVID-19 may reflect the impact of increasingly limited access to appropriately low-phosphorous food options during the initial wave of the pandemic. Specifically, a diet higher in processed and shelf-stable foods, which are more readily accessible and affordable, is likely to be higher in phosphorous content [10]. However, other pandemic related factors may have also influenced phosphorous levels as children not eating school lunches, restaurant closures, and families spending more time indoors and isolating [9]. While hospital related factors may have also contributed, none are readily identifiable to the authors. Neither hospital had shortage of dialysis equipment, delays in dialysis treatment, or reduced hours of dialysis treatment per patient during the pandemic. There are too many variables that impact phosphorous intake to draw a conclusion from this finding. We note that long-term increased serum phosphorus levels are associated with severe clinical consequences including cardiovascular tissue calcification and chronic kidney diseasemineral bone disease and hyperparathyroidism, leading to increased cardiovascular disease and mortality rates.

Additionally, we report lower rates of FI at Seattle Children’s Hospital compared to Riley Hospital for Children, as well a lower rate of FI than that reported in a previous FI study at Seattle Children’s among a different clinical cohort of ESKD patients [4]. We speculate that these differences may potentially reflect the positive impact of earlier adoption of FI screening and the availability of an on-site food pantry at Seattle Children’s Hospital, the establishment of which preceded the COVID-19 pandemic. Conversely, no on-site food pantry was available at Riley Hospital for Children during the time of this study. Similar to what has been reported in other patient populations, availability of an on-site food pantry may have decreased barriers to accessing food for families with FI [9, 11, 12]. While no baseline data on FI rates at Riley Hospital for Children is available, we speculate that the lower rates at Seattle Children’s despite the COVID-19 pandemic may reflect the previous work and resources available to this population, particularly the presence of an on-site food pantry. There are a growing number of food as medicine programs which include medical meals, mobile food pantries and home food delivery programs that could provide the benefits of an on-site food pantry without the added expense and coordination required with a food pantry. However, any food as medicine program would have to be carefully calibrated to provide chronic disease appropriate food for each individual patient (e.g. phosphorus, potassium and sodium restrictions), and furthermore, availability of such programs is highly variable by region.

Both FI and COVID-19 are known to disproportionately affect racial minorities, and structural racism is an important underpinning of these disparities [13]. While we did not observe differences in the prevalence of FI by race, this may have been due to small sample size. Additionally, we suspect that differences may exist in FI based on modality of dialysis, however given our small sample size we are unable to evaluate this difference. This should be further evaluated in future work. A recent cross-sectional study of national data found that during the COVID-19 pandemic, FI did not significantly differ between racial groups, but racial minorities were significantly less confident about their food security compared to white participants [14].

Food insecurity is an essential social determinant of health that has been tied to higher rates of chronic disease and poorer health outcomes [15]. These concerns are exacerbated by a global pandemic that has highlighted disparities and further strained already-limited social resources. Increased rates of unemployment and poverty, two strong drivers of FI, increased worldwide following the COVID-19 pandemic [16]. Other factors which may contribute to high FI prevalence during COVID-19 include school closures and virtual learning resulting in the loss of SNAP subsidized meals [17, 18].

This study has several limitations. First, patients and families may have reasons not to disclose FI, thus leading to an underestimate of FI prevalence. Enlisting the help of a social worker or other dialysis team member with good rapport with families may help them to feel more comfortable in disclosing FI [19]. We also note that the Hunger Vital Signs screening questions are designed to capture food insecurity within the last 12 months, and are of limited utility in assessing shorter term food insecurity. Further, owing to the relative rarity of pediatric ESKD, the sample size is limited. Additionally, we do not have available data on the prevalence of FI at Riley Hospital for Children prior to the COVID-19 pandemic, limiting longitudinal comparions among part of the cohort. Despite these limitations, our study highlights the frequency and importance of FI among children with ESKD.

## Conclusion

Our findings support the implementation of routine assessment of FI in all children with ESKD, especially during periods of high community stress, such as the COVID-19 pandemic. Identification of FI through frequent screening, and subsequently developing targeted interventions such as access to on-site food banks with diet-appropriate foods, offers the possibility of improving outcomes for these children. Future studies need to evaluate the clinical outcomes of these interventions.

## Supplementary Information

**Additional file 1: Supplemental Table 1.** Patient characteristics stratified by Food Security Status^1^.

**Additional file 2: Supplemental Table 2.** Baseline patient characteristics stratified by Site^1^.

**Additional file 3: Supplemental Table 3. **Markers of Nutritional Status during COVID pandemic stratified by Food Security Status.

**Additional file 4: Supplemental Table 4. **Markers of Nutritional Status prior to and during COVID pandemic among food insecure and food secure children with ESKD.

## Data Availability

The datasets used and/or analysed during the current study are available from the corresponding author on reasonable request.

## Abbreviations

COVID-19: Coronovirus 2019
ESKD: End-Stage Kidney Disease
FI: Food Insecurity
FSGS: Focal Segmental Glomerulosclerosis
HD: Hemodialysis
IQR: Inter-Quartile Range
KDIGO: Kidney Disease Improving Global Outcomes
PD: Peritoneal Dialysis
SD: Standard Deviation
SNAP: Supplemental Nutrition Assistance Program
