# Supplementary material for: Food insecurity during COVID-19 in children with end-stage kidney disease: a pilot study
Source: BMC Pediatr. 2022 Jul 15;22:420. doi: 10.1186/s12887-022-03472-2 (PMC9284949; doi:10.1186/s12887-022-03472-2)
Supplement: Supplementary file 1 — Additional file 1: Supplemental Table 1. Patient characteristics stratified by Food Security Status1. [file 12887_2022_3472_MOESM1_ESM.docx]

**Supplemental Table 1**. Patient Characteristics Stratified by Food Security Status^1^

|  | Food Insecure (*n*=18) | Food Secure (*n*=11) | All Participants  (*n*=29) |
| --- | --- | --- | --- |
| Male  Age (years), Mean (SD)  Race  American Indian/Alaska Native  Black  Caucasian  Other/Unknown  Reason for ESKD  Glomerulonephritis  Cystic kidney disease  Renal dysplasia  Obstructive uropathy  FSGS  Congenital nephrotic syndrome  Other | 12 (67%)  12.9 (4.5)  2 (11%)  7 (39%)  6 (33%)  3 (17%)  4 (22%)  0  5 (28%)  3 (17%)  3 (17%)  0  3 (17%) | 4 (36%)  9.4 (6.1)  0  4 (36%)  5 (45%)  2 (18%)  1 (9%)  1 (9%)  3 (27%)  2 (18%)  1 (9%)  1 (9%)  2 (18%) | 16 (55%)  11.6 (5.32)  2 (7%)  11 (38%)  11 (38%)  5 (17%)  5 (27%)  1 (3%)  8 (28%)  5 (17%)  4 (14%)  1 (3%)  5 (17%) |
| Dialysis Modality  Hemodialysis  Peritoneal Dialysis | 17 (94%)  1 (6%) | 8 (73%)  3 (27%) | 25 (86%)  4 (14%) |

COVID-19, Coronavirus Disease 19; ESKD, End-Stage Renal Disease; FSGS, Focal Segmental Glomerulosclerosis; SD, Standard Deviation.

^1^Between-group comparisons by chi-square test or Fisher exact test and Wilcoxon rank-sum for categorical and continuous variables, respectively.
