# Supplementary material for: Food insecurity during COVID-19 in children with end-stage kidney disease: a pilot study
Source: BMC Pediatr. 2022 Jul 15;22:420. doi: 10.1186/s12887-022-03472-2 (PMC9284949; doi:10.1186/s12887-022-03472-2)
Supplement: Supplementary file 2 — Additional file 2: Supplemental Table 2. Baseline patient characteristics stratified by Site1. [file 12887_2022_3472_MOESM2_ESM.docx]

**Supplemental Table 2**. Baseline Patient Characteristics Stratified by Site^1^

|  | Riley/IU (*N*=14) | SCH (*N*=15) | Total (*N*=29) |
| --- | --- | --- | --- |
| Male  Age (years), Mean (SD)  Race  American Indian/Alaska Native  Black  Caucasian  Other/Unknown  Reason for ESRD  Glomerulonephritis  Cystic kidney disease  Renal dysplasia  Obstructive uropathy  FSGS  Congenital nephrotic syndrome  Other | 11 (79%)  11.7 (4.11)  0  6 (43%)  8 (57%)  0  2 (14%)  0  5 (36%)  3 (21%)  1 (7%)  0  3 (21%) | 5 (33%)  11.4 (6.29)  2 (13%)  3 (20%)  5 (33%)  5 (33%)  3 (20%)  1 (7%)  3 (20%)  2 (13%)  3 (20%)  1 (7%)  2 (13%) | 16 (55%)  11.6 (5.32)  2 (7%)  9 (31%)  13 (49%)  5 (17%)  5 (27%)  1 (3%)  8 (28%)  5 (17%)  4 (14%)  1 (3%)  5 (17%) |
| Dialysis Modality  Hemodialysis  Peritoneal Dialysis | 14 (100%)  0 | 11 (73%)  4 (27%) | 25 (86%)  4 (14%) |
| Food Insecurity | 12 (86%) | 6 (40%) | 18 (62%) |

COVID-19, Coronavirus Disease 19; ESRD, End-Stage Kidney Disease; FSGS, Focal Segmental Glomerulosclerosis; SD, Standard Deviation; SCH, Seattle Children’s Hospital

^1^Between-group comparisons by chi-square test or Fisher exact test and Wilcoxon rank-sum for categorical and continuous variables, respectively.
