# Supplementary material for: Food insecurity during COVID-19 in children with end-stage kidney disease: a pilot study
Source: BMC Pediatr. 2022 Jul 15;22:420. doi: 10.1186/s12887-022-03472-2 (PMC9284949; doi:10.1186/s12887-022-03472-2)
Supplement: Supplementary file 3 — Additional file 3: Supplemental Table 3. Markers of Nutritional Status during COVID pandemic stratified by Food Security Status. [file 12887_2022_3472_MOESM3_ESM.docx]

**Supplemental Table 3.** Markers of Nutritional Status During COVID Pandemic Stratified by Food Security Status

|  | Food Insecure (*n*=18) | Food Secure (*n*=11) | *p* value |
| --- | --- | --- | --- |
| Phosphorus (median, IQR) | 6.7 (2.1) | 6.0 (2.1) | 0.57 |
| Phosphorus in goal range (percent) | 2 (11%) | 4 (36%) | >.99 |
| Phosphorus Delta (median, IQR) | 1.1 (1.3) | 0 (2) | 0.03 |

Phosphorus reported in mg/dL
