# Supplementary material for: Food insecurity during COVID-19 in children with end-stage kidney disease: a pilot study
Source: BMC Pediatr. 2022 Jul 15;22:420. doi: 10.1186/s12887-022-03472-2 (PMC9284949; doi:10.1186/s12887-022-03472-2)
Supplement: Supplementary file 4 — Additional file 4: Supplemental Table 4. Markers of Nutritional Status prior to and during COVID pandemic among food insecure and food secure children with ESKD. [file 12887_2022_3472_MOESM4_ESM.docx]

**Supplemental Table 4**. Markers of Nutritional Status Prior to and During COVID Pandemic Among Food Insecure and Food Secure Children with ESKD

|  | Baseline | During COVID | *p* value |
| --- | --- | --- | --- |
| Food Insecure (*n*=18)  Phosphorus (median, IQR)  Phosphorus in goal range (percent) | 5.3 (2.1)  9 (50%) | 6.7 (2.1)  2 (11%) | 0.04  0.03 |
| Food Secure (*n*=11)  Phosphorus (median, IQR)  Phosphorus in goal range (percent) | 5.5 (2.7)  4 (36%) | 6.0 (2.0)  4 (36%) | 0.32  1.00 |

Phosphorus reported in mg/dL
